# Cases in Practice

**Published:** 1875-09

**Authors:** F. K. Bailey

**Affiliations:** Knoxville, Tenn.


### CASES IN PRACTICE.

### I. POST PARTUM HEMORRHAGE—SUSPECTED LACERATION OF THE CERVIX UTERI.

By F. K. Bailey, M. D., Knoxville, Tenn.

July 23, 1874.—Called in consultation with Dr. Kuaffe, of this city, at 8 o'clock a. m., to see a German lady about 35; multipara. Good previous health, and at full term. Was taken about 4 a. m. with pains, when a midwife was called. Labor progressed very rapidly, and was finished with fewer pains than usual for her. Midwife reports that the placenta came away at once, but soon profuse flooding commenced, and Dr. K. called in. He found the womb contracted and in situ, and at once attributed the hemorrhage to some unusual cause. On passing the hand into the vagina, he found it widely distended, with blood firmly coagulated. The coagula being removed, he passed his hand so as to reach the fundus uteri without any trouble. On exploring the os, he found that on the left side of the cervix there was a point where no resistance offered—there was a laceration.

The woman complained of severe pain, and was very much prostrated. He gave opium, and applied cold, wet cloths to the pubic region. On my arrival, her face was deathly pale, pulse small and weak, and she was free from pain. On passing two fingers carefully within the vagina so as to reach the os, I found the right side feeling natural, but upon the left, instead of the normal continuity of structure, there was a sulcus filled with coagulum. Did not disturb the clot, but considered it certain that a laceration had occurred. We waited thirty minutes, and finding that reaction was coming up, left the house, giving instructions to apply cold to the pubis, and to give wine with a little food.

5 p. m.—Reaction has come up, with no increase of frequency of the pulse. An occasional twinge of pain through the uterine region, which is considered after-efforts at contraction.

24th, 12 M.—Dr. K. informs me that his patient is very comfortable to-day. No fever. He drew off a moderate quantity of urine last night. Slight after-pains, and tochia normal in quantity and quality. Is using a carbolised solution per vaginam.

25th.—Doing well. Some milk appearing.

27th.—Calling in, I found the lady doing well. Pulse 100. Milk in abundance. Lochia not copious, but normal. Considerably prostrated, but the skin is cool and moist.

[The recovery was complete.]

May, 1875.

II. HEMORRHAGE FROM THE MOUTH, AND OTHER MUCOUS SURFACES.

April 28, 1875.—Called at 10 A. M. to see Jane T——, a colored woman of 45; mother of 15 children; 6 living.

Has been irregular for some months in menstruating, with profuse discharge at times, and leucorrhœa constant. Bowels costive. Yesterday, was taken with bleeding from the gums and mouth generally. Says she lost half a gallon or more.

The pulse is weak, and there is evident depression.

Some vaginal discharge of bloody mucus, not attended with much pain in the pelvic region.

As I sat by the bedside, blood was flowing moderately from the mouth, and also some from the nose.

Gave at once xv. Fluid Ext. Ergot, to be repeated every three hours during the day, and prescribed as follows, viz:

℞.—Bromide Potassii, . . . ʒ ij.

Fluid Ext. Sec. Connenti, ℥ ss.

Sqr. Pruni Virgine, . . ℥ iss.

M. Sig. Teaspoonful three times daily.

6 P. M.—No change for the better. Nose bleeding more profusely, and flowing also from the vagina.

April 29.—The husband reports that hemorrhage appeared to cease, in part from the mouth and nose, but still continued from the vagina. Not being able to visit her in the morning, directed a continuance of the mixture, with whisky punch to obviate faintness.

30th.—Died last night from exhaustion.

I am told that in August last this woman menstruated regularly for the last time. Nothing very definite could be learned in regard to details.

The past winter and spring have been very trying to females of every age in the lower classes. Long continued damp and cool weather had the effect to cause derangements in the menstrual function. Rheumatism and neuralgia have been common, and determination of blood to the pelvic organs unusually so. In the case just described, a hèmorrhagic condition obtained, and fatal loss of blood rapidly obtained.

I am of the opinion that ergot might have proved beneficial, if administered at an early period of her sickness.

### III. THREATENING SYMPTOMS FROM A PUNCTURED WOUND.

March 31, 1874.—Was called in to see W. T. M——, a stout, well-built man of 65 or more, who had a few days previously stepped upon a couple of rusty nails, causing puncture at the middle of the ball of the left foot, about an inch apart. The thickened cutis was torn, and the cellular tissue penetrated about a quarter of an inch. There was swelling, some heat and great tenderness in the sole of the foot over an area of three inches or more. There was a tendency to contraction of the extensor muscles of the second and third toes—"a cramping sensation." A thin ichorous discharge flowed from the punctures. There is a discolored spot three inches in extent upon the anterior aspect of the same limb, just above the ankle, caused by an injury received twenty-five years ago. At this point there has been at times ever since an ulcer, which discharged freely, of an unhealthy pus. There is a varicase condition of the veins upon the leg, and, upon the whole, an unpromising state of things.

Apprehending tetanus, I at once introduced to the openings a strong solution of carbolic acid in glycerine—equal parts of each, and water six parts. This caused intense smarting. Ordered a warm poultice to be applied, and a dose of epsom salts taken, at once. Dover's powder at bed-time.

April 1, 9 A. M.—Find the foot still intensely sore, with increased swelling. The smarting from carbolic acid lotion continued till 2 A. M. Bowels open. No arterial excitement, and appetite good. Enlarged the punctures with a bistoury, and reapplied the lotion. Directed the limb to be kept in a horizontal position on a pillow, or on the bed.

4 P. M.—Swelling still extending, and the parts intensely sensitive. Cramping of the toes continues. Some erysipelatous blush appearing, I applied strong solution carbolic acid, and prescribed the following mixture:

℞.—Tr. Iodine, . . . . . . ℥ ss.

Glycerine, }

Tr. Opii, } . . . . . aa ʒ ij.

Carbolic Acid, . . . . . ℈ss.

M. Sig. Apply freely every 2 or 3 hours.

April 2, 8 A. M.—Find the foot much improved; swelling diminished, and less tenderness. Cannot set his foot to the floor without increased pain, but there is no more contraction of the extensors. After freely incising the parts affected, introduced strong solution carbolic acid; to continue the lotion, and apply warm poultices. The man informs me to-day that on a previous occasion he injured the same foot, and there were strong indications of lockjaw.

3d, 10 A. M.—Much better. Entire freedom from pain when the limb is kept in a horizontal position. A painful fullness is felt as soon as the foot is lowered to the floor. To continue the iodine lotion, and keep quiet.

4th.—Still improving. Continue lotion.

10th.—The puncture nearly healed. Has been out for two days, but continned to apply the lotion.

Tetanus often follows such wounds as the above, and in this case there were serious apprehensions. The anticeptic course appeared to act like a charm to obviate the accession of erysipelas even if tetanus did not threaten.

